# Unlocking the secrets of ABEs: the molecular mechanism behind their specificity

**DOI:** 10.1042/BST20221508

**Published:** 2023-08-01

**Authors:** Xiaoyu Chen, Mitchell J. McAndrew, Audrone Lapinaite

**Affiliations:** 1 School of Molecular Sciences, Arizona State University, Tempe, AZ, U.S.A.; 2 Arizona State University-Banner Neurodegenerative Disease Research Center at the Biodesign Institute, Arizona State University, Tempe, AZ, U.S.A.; 3 Center for Molecular Design and Biomimetics, The Biodesign Institute, Arizona State University, Tempe, AZ, U.S.A.

**Keywords:** deamination, DNA adenine base editor, DNA base editing, DNA deaminase, genome editing, tRNA deaminase

## Abstract

CRISPR–Cas, the bacterial immune systems, have transformed the field of genome editing by providing efficient, easily programmable, and accessible tools for targeted genome editing. DNA base editors (BE) are state-of-the-art CRISPR-based technology, allowing for targeted modifications of individual nucleobases within the genome. Among the BEs, adenine base editors (ABEs) have shown great potential due to their ability to convert A-to-G with high efficiency. However, current ABEs have limitations in terms of their specificity and targeting range. In this review, we provide an overview of the molecular mechanism of ABEs, with a focus on the mechanism of deoxyadenosine deamination by evolved tRNA-specific adenosine deaminase (TadA). We discuss how mutations and adjustments introduced via both directed evolution as well as rational design have improved ABE efficiency and specificity. This review offers insights into the molecular mechanism of ABEs, providing a roadmap for future developments in the precision genome editing field.

## Introduction

A single nucleotide variant (SNV, or point mutation) is a substitution of an individual nucleotide in genomic DNA. SNVs not only cause the majority of human genetic diseases [1], but are also associated with desirable traits in crops and livestock [2,3]. Thus, there is a pressing need to develop precision genome editing tools able to introduce point mutations in a targeted manner.

Current genome editing approaches are based on CRISPR (Clustered Regularly Interspaced Short Palindromic Repeats) systems: adaptive prokaryotic immune systems that use guide RNAs (gRNAs, also called crRNAs) to direct effector proteins (usually nucleases) to inactivate foreign nucleic acids [4–6]. Based on effector-guide RNA complex composition, CRISPR systems are classified into class I or II, with additional division into different types. Class II CRISPR systems feature an effector complex composed of a single protein and crRNA and include Type II and Type V systems, with Cas9 and Cas12 effector proteins, respectively. They are the preferred platforms for engineering genome editing tools because they can be easily programmed to introduce double-stranded DNA breaks (DSBs) at the specific genomic DNA loci [7–10]. In cells, these DSBs are then repaired via one of the following DNA repair pathways: homology-directed repair (HDR), non-homologous end joining (NHEJ), or polymerase theta-mediated end joining (TMEJ) [8,9,11–13]. HDR is the only pathway that introduces precise DNA modifications; however, it is limited to actively dividing cells and requires a donor DNA template (reviewed in [14]). These drawbacks hinder the application of CRISPR–Cas tools for precision genome editing. However, CRISPR-based DNA base editing offers a solution by enabling precision DNA modifications without the need for DSBs or HDR.

**Figure 1.**
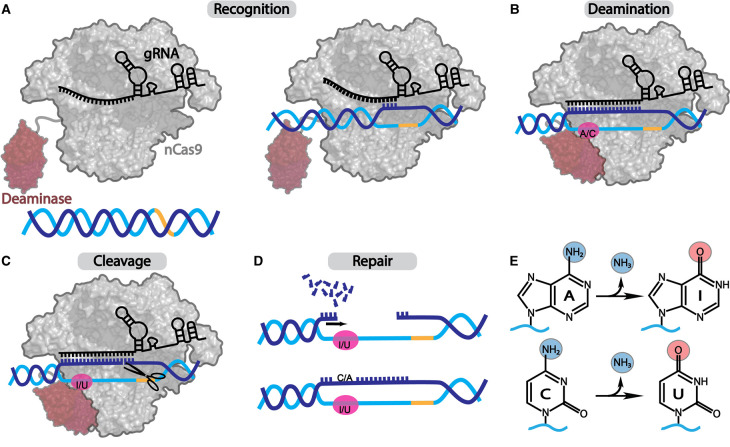
General process of DNA base editing. DNA base editing is achieved via four events: (**A**) Recognition: BE RNPs locate appropriate PAM site (yellow) in the genome, unwind the dsDNA to verify DNA complementarity to the gRNA, and if successful, form a stable R-loop; (**B**) Deamination: the tethered deaminase interacts with the exposed ssDNA bases in the editing window (pink) of the R-loop and catalyzes the deamination reaction; (**C**) Cleavage: Cas effectors (typically nCas9) cleaves the unedited TS; (**D**) Repair: the nicked strand in genomic DNA triggers the SSB repair pathway, which uses the edited strand as a template for repair, resulting in the incorporation of the modification into both strands of the DNA. (**E**) Schematic representation of the deamination reaction catalyzed by ABEs (top, dA-to-dI editing) and CBEs (bottom, dC-to-dU editing).

DNA base editors (BEs) are designed to introduce targeted point mutations in genomic DNA [15,16]. There are two main types of BEs: cytosine base editors (CBEs; converting C-G base pairs to T-A) and adenine base editors (ABEs; converting A-T base pairs to G-C). CRISPR–Cas9-based ABEs consist of an RNA-guided catalytically impaired nickase Cas9 (nCas9: is able to cut one strand of the target DNA) fused to a laboratory-evolved bacterial tRNA adenosine deaminase (TadA) [16–18]. The nCas9-guide RNA module locates a target site and nicks the target strand (TS) in genomic DNA, while the engineered TadA deaminase catalyzes the conversion of deoxyadenosine (dA) to deoxyinosine (dI) in single-stranded DNA (ssDNA) within the R-loop (a structure formed by gRNA hybridization with one DNA strand, while displacing the opposing strand) through a deamination reaction. The dI is then recognized by cellular DNA repair machinery as deoxyguanosine (dG), resulting in the conversion of an **A**-T base pair to a **G**-C base pair (Figure 1). CBEs have a similar composition and mechanism to ABEs, but instead of TadA, a cytosine deaminase domain is used (often an engineered version of mRNA cytosine deaminase APOBEC1) to deaminate deoxycytidine (dC) to deoxyuridine (dU) [15]. The resulting dU is recognized by cellular repair machinery as deoxythymidine (dT), leading to the conversion of a **C**-G base pair to a **T**-A base pair.

Various versions of BEs have been successfully used to generate targeted point mutations in the genomic DNA of multiple organisms and treat T-cell acute lymphoblastic leukemia in a single patient [19–24]. However, current BEs have limitations including off-target DNA and RNA editing and high-efficiency bystander editing (when neighboring nucleotides to the target nucleotide are edited), which may lead to undesired genomic changes. The molecular mechanisms that explain the efficiency and specificity of DNA base editing, and the basis of BEs’ shortcomings remain poorly understood. Since the only available structure of a full-length BE in complex with its target DNA is one of the ABE (specifically, ABE8e) [25], here, we review recent research that broadens our understanding of how ABEs modify their on- and off-targets. We discuss how this knowledge guides future design efforts for more efficient and specific ABEs suitable for various applications in the fields of biomedicine, agriculture, and climate change.

## Molecular basis of DNA deoxyadenosine deamination

**Figure 2.**
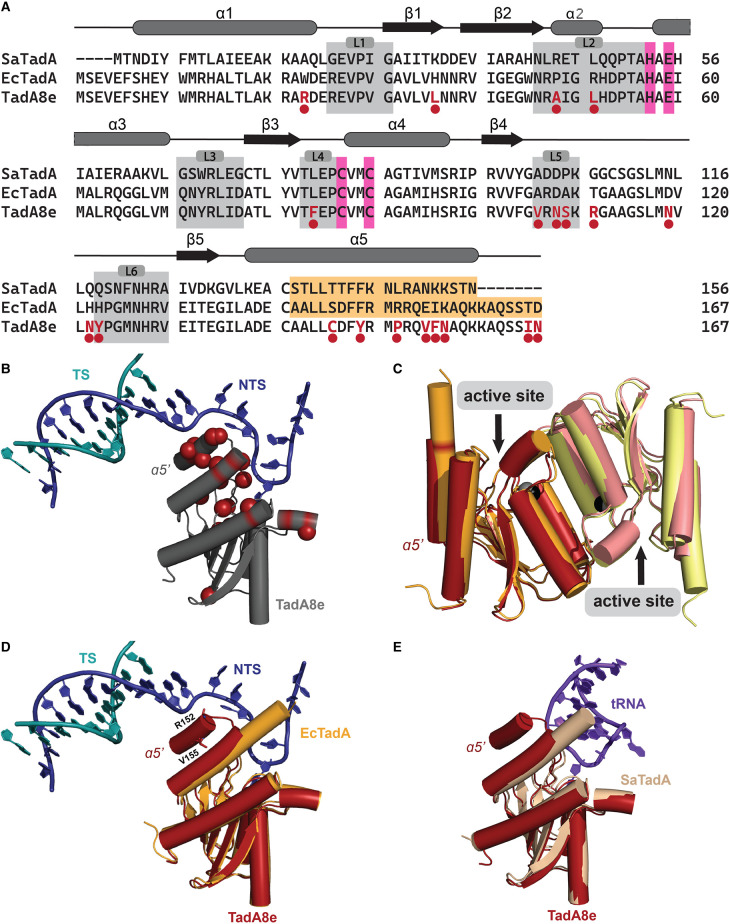
Sequence and structural comparisons of natural tRNA and evolved DNA deaminases. (**A**) Alignment of SaTadA, EcTadA, and TadA8e sequences with assigned secondary structure elements on the top (α-helices as gray ovals; β-strands as black arrows). The amino acids mutated during directed evolution are highlighted in red and marked with a circle. Gray boxes signify the substrate-binding loops (L1–L6). The orange box highlights the residues of α5-helix that interact with and stabilize tRNA in the binding pocket of SaTadA and EcTadA. Catalytic residues are highlighted in pink boxes. (**B**) The substitutions introduced during directed evolution (red spheres) are mapped onto the structure of a single TadA8e domain in complex with the DNA substrate (gray, PDB ID: 6VPC). (**C**) The aligned structures of EcTadA (orange and yellow, PDB ID: 1Z3A) and TadA8e (red and pink, PDB ID: 6VPC) exhibit an overall similar 3D shape, with the only observed difference being in the conformation of the α5-helix. Both enzymes form homodimers via the same dimerization interface positioning their active sites on opposite sides. The aligned structures of (**D**) apo EcTadA (orange) and DNA-bound TadA8e (red) and (**E**) tRNA-bound SaTadA (pale-yellow, PDB ID: 2B3J) and TadA8e (red) showing the position of TadA8e's α5′-helix in respect to the DNA and the tRNA substrates, respectively.

**Figure 3.**
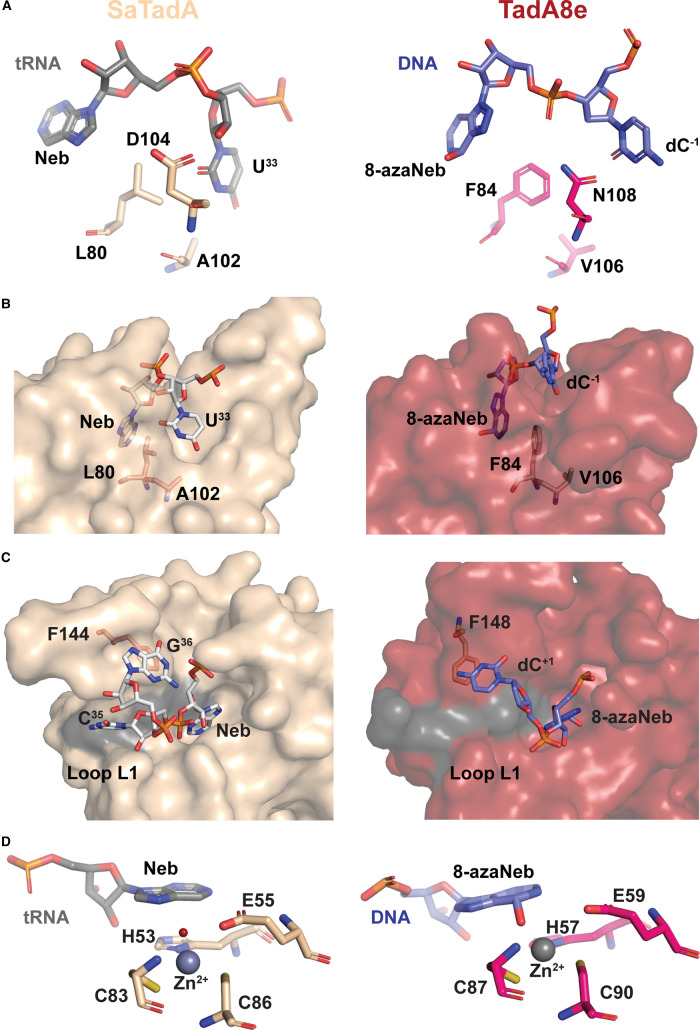
Comparison of interactions between TadA variants and their preferred substrates. (**A**) In SaTadA (left, pale-yellow and gray, PDB ID: 2B3J), the D104 residue forms a hydrogen bond with the 2′-OH group of U^33^ ribose. In TadA8e (right, magenta and blue, PDB ID: 6VPC), the N108 residue (equivalent of D104) is positioned closer to the phosphate group and the base of dC^−1^. (**B**) Comparison of U^33^ and dC^−1^ binding pockets in SaTadA (left) and TadA8e (right), respectively. (**C**) Comparison of C^35^ and dC^+1^ binding pockets in SaTadA (left) and TadA8e (right), respectively. (**D**) Comparison of SaTadA (left) and TadA8e (right) active sites with the Neb and 8-azaNeb instead of the target adenosine, respectively.

Deoxyadenosine base editing in genomic DNA involves four events: (1) recognition of a specific genomic DNA sequence and the formation of an R-loop (Figure 1A), (2) deamination of the target deoxyadenosine in the non-target strand (NTS) of the R-loop (Figure 1B), (3) cleavage of the unedited TS (Figure 1C), and (4) repair of the cut strand to introduce the edit to the unmodified strand (Figure 1D). The Cas9 domain of ABE, in complex with gRNA, drives events 1 and 3. Event 2 is performed by the TadA deaminase domain, and event 4 relies on cellular DNA repair pathways. The order of events 1–3 is currently unknown and likely depends on the enzyme kinetics of the specific variants of Cas effector and deaminase domain used.

### Cas9–gRNA locates genomic DNA sites for base editing

Since the deaminase domain is fused to Cas9 via a long and flexible linker and the cryo-EM structure of ABE8e does not show extensive protein–protein interactions between TadA8e and Cas9 [25], it is reasonable to assume that ABEs’ target-search mechanism is similar to that of Cas9. The search for a genomic target site starts with the formation of a Cas9–gRNA complex (Cas9 RNP), which then locates the protospacer adjacent motif (PAM) in the genomic DNA via both random three-dimensional collision and one-dimensional diffusion [26–28]. For *Streptococcus pyogenes* Cas9 (SpCas9), the PAM is a trinucleotide sequence (5′-NGG) located on the non-target strand, which will not base-pair with the gRNA [29]. After identifying the PAM, Cas9 RNP starts unwinding the double-stranded DNA (dsDNA) to verify DNA complementarity to the gRNA. If successful, a stable R-loop complex forms, where the TS is in duplex with the gRNA while the NTS is in a flexible ssDNA state [30–32], serving as the substrate for the deaminase domain of ABE.

### Evolved tRNA deaminase catalyzes deoxyadenosine deamination in ssDNA

#### Overall structure

*Escherichia coli* and *Staphylococcus aureus* tRNA-specific deaminases (EcTadA and SaTadA, respectively) selectively deaminate adenosine at the wobble position 34 of tRNA^Arg2^. They achieve this specificity by recognizing both the rigid structure of the tRNA anticodon stem and the U^33(−1)^A^34(0)^C^35(+1)^G^36(+2)^ sequence in the anticodon loop [33,34]. The catalytic domain of ABEs (including ABE8e [TadA8e]) was obtained via directed evolution of EcTadA [16,18,35]. TadA8e is the most efficient DNA deoxyadenosine deaminase to date and differs from EcTadA by 20 amino acid substitutions (Figure 2A) [18]. Most of these mutations are found within the substrate-binding loops and the C-terminal α5-helix (Figure 2A,B). These regions establish extensive interactions with the DNA substrate, or in the case of EcTadA and SaTadA, with the tRNA substrate (Figure 2E). Cryo-EM structure of ABE8e in a DNA-bound state [25] and molecular dynamics (MD) simulations [36–38] provide insights into the mechanism of ssDNA deoxyadenosine deamination and explain how amino acids introduced during directed evolution contribute to the new activity of the TadA8e deaminase.

The overall 3D shape of EcTadA and TadA8e is comparable (Figure 2C), which suggests that mutagenesis performed during directed evolution did not result in significant structural changes, but rather focused on optimizing the enzyme's active site and substrate-binding loops. Both deaminases form homodimers through the same dimerization interface (composed of α2, α3 and α4 helices), which is not unexpected since only two amino acid substitutions, H122N and H123Y, are located near the dimerization interface. However, further research is required to determine whether these mutations affect the stability of the homodimer. Nonetheless, current data from native mass spectrometry and cryo-EM structure indicate that TadA8e forms stable homodimers [25]. The structure of SaTadA–tRNA complex (PDB ID: 2B3J, the only structure of bacterial TadA–tRNA complex) demonstrates that the substrate-binding pocket consists of both monomers (same is observed for TadA8e) [25,39] and suggests that dimerization might be necessary for efficient tRNA deamination. However, the significance of TadA dimerization in either tRNA or DNA deamination has yet to be demonstrated.

It is important to note that some studies refer to ABEs as monomeric when one TadA domain is fused to Cas9 and as dimeric when two TadA domains are fused to Cas9 [16,18]. In both cases, the intact dimerization interface facilitates the formation of dimeric ABEs through TadA dimerization, occurring either in *trans* or in *cis* [25]. The role of ABE dimerization in genomic DNA editing remains unclear and requires further investigation.

#### Interactions with the phosphate backbone

Upon interaction with TadA8e, the flexible ssDNA substrate acquires a U-turn conformation, similar to the tRNA anticodon loop bound by SaTadA [25,39]. The ssDNA phosphate backbone is likely held in place by interactions similar to those seen in the SaTadA–tRNA complex. The tRNA loop is stabilized by positively charged side chains: R44, K106 and R149 (monomer A), and R70 and R94 (monomer B) in SaTadA [34,39]. In EcTadA, equivalent residues are P48, K110, and R153 (monomer A), and R74 and R98 (monomer B) [34,39]. These amino acids, with the exception of P48, were not mutated in TadA8e and are likely to establish similar interactions with the ssDNA backbone, especially with the phosphate groups of 8-azanebularine (8-azaNeb), dC^+1^, and dT^+2^, which overlap well with tRNA residues 33–35 [25].

TadA8e forms additional interactions with the ssDNA backbone due to mutations introduced during directed evolution. Specifically, the side chain of T111R is likely to interact with the phosphate group of 8-azaNeb [25,36]. Its importance is evident from biochemical assays showing near complete loss of DNA deamination when mutated back to threonine [25]. Another source of new interactions are mutations found in the α5-helix. EcTadA ɑ5-helix is straight, while in TadA8e it exhibits a noticeable bend of ∼25° attributed to the R152P mutation and possibly reinforced by the hydrophobic E155V residue (Figure 2A,D) [25]. The SaTadA–tRNA structure reveals that the α5-helix stabilizes the conformationally remodeled tRNA by interacting with its major groove [39] (Figure 2E). These interactions are lost in TadA8e. Instead, the α5-helix points towards the 5′-end of the NTS and guides the flexible ssDNA to the active site of TadA8e (Figure 2D) [25]. The α5-helix orientation suggests that R153 and K157N likely interact with the ssDNA backbone. The significance of the α5-helix in substrate recognition and ssDNA deamination is highlighted by eight out of the 20 mutations concentrated in this region, with some introduced in the initial rounds of directed evolution (Figure 2A,B) [16,18].

#### Discrimination between RNA and DNA substrates

D108N is a critical mutation that appeared in the first round of directed evolution and yielded ABE1.1 with detectable DNA deamination activity [16,36]. This substitution brings about an interesting change — MD simulations indicate that this substitution causes the β4–β5 loop to become more rigid [38]. Given that this loop contains two ligand binding regions (L5 and L6, Figure 2A), the mutation at position 108 holds significance for substrate interactions. The SaTad–tRNA structure demonstrates that D104 (D108 in EcT108adA) forms a hydrogen bond with the 2′-OH group of U^33^ ribose (Figure 3A) [39]. However, in the case of TadA8e, this interaction is disrupted due to the altered phosphate backbone trajectory of the flexible ssDNA substrate: the sugar moiety of dC^−1^ points away from the N108 residue, while the phosphate group of 8-azaNeb positions itself closer to N108 (Figure 3A) [25]. Thus, the substitution of the negatively charged D with the polar N allows TadA8e to accommodate ssDNA substrates in this conformation and discriminate it from rigid tRNA anticodon stem loops [25]. Interestingly, there are natural bacterial TadA enzymes that have asparagine at this position [37,40], suggesting that some TadA enzymes could in principle naturally deaminate ssDNA.

The fact that SaTadA interacts with only one 2′-OH group suggests that the enzyme's substrate recognition is likely governed more by the tRNA's 3D shape and sequence than by interactions with 2′-OH groups. The absence of this single interaction in evolved TadA enzymes may not be sufficient for them to differentiate between DNA and RNA substrates. This is supported by *in vitro* and cell-based assays showing that all TadA-based ABEs deaminate not only DNA but also RNA [17,18,25]. Thus, the selective pressure used to drive the evolution of TadA-based ABEs has led to enzymes that differentiate between rigid, structured substrates and flexible, single-stranded ones, rather than distinguishing between DNA and RNA.

#### Sequence recognition

SaTadA and EcTadA have the same sequence preference: the U^33(−1)^A^34(0)^C^35(+1)^G^36(+2)^ sequence in the anticodon loop [34,39]. In SaTadA, U^33^ (dC^−1^ for TadA8e) is nestled within a partially open groove and recognized via two hydrogen bonds — one between S138 (or A142 in EcTadA) and the carbonyl group at C4, and another between D103 (or R107 in EcTadA) and N3 of U^33^. TadA8e lacks these interactions as A106V and L84F mutations alter the binding pocket for U^33^ (dC^−1^). These bulky and hydrophobic side chains push dC^−1^ away from its usual position (U^33^ would occupy), making the binding pocket shallower (Figure 3B). This explains TadA8e's slight preference for smaller pyrimidine bases at the −1 position [41]. Moreover, the recognition of U^33^ in tRNA by SaTadA and EcTadA relies on two key residues, S138 and D103 in SaTadA, and A142 and R107 in EcTadA, located in the α5-helix. The R152P mutation in TadA8e alters the direction of the α5-helix and disrupts these interactions. This mutation was introduced to ABE during the seventh round of evolution and it coincides with the loss of sequence specificity [16].

To trap TadA structures in a substrate-bound state, the target adenosine is replaced with adenosine analogs like nebularine (Neb) or 8-azaNeb to mimic the transition state of the deamination reaction [42,43]. TadA8e likely recognizes 8-azaNeb in a similar manner to SaTadA's recognition of Neb, as both nucleotides are in similar position in their respective structures [25,39].

In SaTadA, the base C^35(+1)^ is relatively solvent-exposed and forms a hydrogen bond with the backbone carbonyl of G22 (R26 in EcTadA). There are no base-specific contacts, suggesting that SaTadA may have flexibility at this position. By comparing the apo EcTadA and tRNA-bound SaTadA structures, we observe that the C^35^ binding pocket in EcTadA is smaller due to the presence of R26 and R152 residues. Their side chains may also form base-specific contacts with C^35^, explaining why EcTadA cannot accommodate other nucleobases at this position [33]. However, a tRNA-bound structure of EcTadA is needed to confirm this hypothesis. In TadA8e, the nucleobase of dC^+1^ is away from the substrate-binding loop, L1, and stabilizes F148 via stacking interactions (Figure 3C). As a result, there are no strong interactions between TadA8e and dC^+1^, which allows TadA8e to modify substrates with any nucleotide at this position.

In SaTadA, the nucleotide G^36(+2)^ is folded back into the interior of the tRNA loop and stabilized by F144 (F148 in EcTadA) (Figure 3C). The exocyclic amine of G^36^ forms a hydrogen bond with the phosphate group of Neb^34^, further stabilizing its conformation. The strict preference for G at this position is likely due to base-specific contacts formed by R149 (R153 in EcTadA) [39]. TadA8e lacks the G^36(+2)^ binding pocket due to the altered α5-helix conformation, resulting in no preference for a particular nucleotide at this position [18,25].

TadA8e, with its minimal preference for pyrimidines at the −1 position (YA) [44], is essentially a DNA deaminase that lacks strict sequence specificity. This aligns with the initial concept of ABEs, aiming to create a DNA deaminase capable of targeting deoxyadenosine in any sequence context. Originally, it was an appealing idea to have a universal deaminase for any sequence, but it has become problematic due to extensive bystander editing by processive deaminases such as TadA8e.

#### Catalytic mechanism

The active site of SaTadA features a zinc ion coordinated to H53, C83, and C86, (H57, C87 and C90 in EcTadA and TadA8e) and a zinc-bound water that likely participates in the hydrolysis reaction **(**Figure 3D). E55 (E59 in EcTadA and TadA8e) is the catalytic residue, which is proposed to activate the zinc-bound water and facilitate nucleophilic attack on the C6 atom of the adenine base [34,45]. The ABE8e cryo-EM structure indicates no significant difference between the active sites, thus, the catalytic mechanism of deoxyadenosine and adenosine deamination in DNA and tRNA, respectively, is likely to be conserved [25,34]. However, further research is needed to confirm the DNA deoxyadenosine deamination mechanism.

### Catalytically impaired Cas9 cleaves the unedited DNA strand

While TadA8e deaminates deoxyadenosine within the R-loop, the second strand, which is in a DNA:RNA duplex, remains unmodified and is cleaved by a catalytically impaired Cas9 (e.g. SpCas9 D10A variant, common for BEs). The mechanism of TS cleavage in ABEs is likely the same as that of Cas9 and is reviewed in detail here [46].

### Cellular DNA repair pathways repair the cut strand and introduce the edit to the unmodified strand

During DNA base editing, the Cas9-induced cleavage of the unedited strand creates a single-stranded break (SSB) in the genomic DNA. Its repair is likely to follow the canonical SSB repair pathway. The possible DNA repair pathways activated during ABE-mediated DNA base editing and their impact on the final genome editing product are discussed in detail here [47].

## Challenges and limitations of current ABEs

ABEs have a wide range of applications, but their precision and specificity are limiting factors, due to the risk of off-target editing. Therefore, it is necessary to understand the types of off-target editing that can occur and their underlying molecular mechanisms. Currently, there are two types of off-target effects associated with ABEs: DNA and RNA off-target editing.

### DNA off-target editing

#### Cas-dependent DNA off-target editing

Cas-dependent DNA off-target editing can be classified into two categories: gRNA-dependent or -independent. gRNA-dependent off-targets arise primarily due to Cas effectors’ tolerance for mismatches in the gRNA-target DNA heteroduplex. Algorithms have been developed to predict off-target sites for a particular gRNA–Cas effector complex. These types of off-target effects are well-researched and reviewed in detail here [48]. The proposed strategies to mitigate these off-target effects include engineering the gRNAs [49] or Cas-effectors. Through structure-guided engineering of SpCas9, several high-fidelity SpCas9 effectors have been designed, and incorporated into ABEs [including but not limited to enhanced Cas9 (eCas9) [50,51], high-fidelity Cas9 (Cas9-HF) [51], hyper-accurate Cas9 (HypaCas9) [51], evolved Cas9 (evoCas9) [51], highly enhanced fidelity Cas9 (HeF) [52], and SuperFi-Cas9 (SuperFi) [52]). The resulting ABEs had reduced DNA off-target editing [53,54], however, in most cases, achieving high fidelity comes at the cost of reduced efficiency of the gRNA–Cas complex [52,55,56].

gRNA-independent off-targets are usually observed if the deaminase domain has a high deamination rate. While searching for PAM sequences, Cas9 bends the dsDNA, which causes transient local melting of dsDNA [32], thereby providing an opportunity for fast deaminases to edit the exposed ssDNA. For instance, ABE8e displays gRNA-independent off-target editing to a greater extent than earlier versions (ABE7.10, miniABEmax) that deaminate at 500- to 1000-fold lower rates [18,25,57]. Identifying and predicting gRNA-independent off-targets is more challenging than gRNA-dependent editing because it is difficult to distinguish spontaneous mutations from off-target mutations induced by ABEs in a gRNA-independent manner. Current strategies are reviewed in detail here [58]. Efforts to minimize gRNA-independent off-target editing have primarily focused on rational engineering of the deaminase domain, resulting in variants with overall reduced DNA deamination efficiency and rates. For example, to mitigate DNA off-target editing in ABE8e, a V106W mutation was introduced. This modification decreases the size of the dC^−1^ (U^33^) binding pocket even further (Figure 3B), thereby reducing TadA8e's affinity for the substrate and significantly decreasing overall DNA and RNA editing [18,25].

#### Cas-independent DNA off-target editing

Cas-independent DNA off-target editing can be classified into two categories: random editing of ssDNA and bystander editing. Lapinaite et al. [25] revealed that TadA8e can edit ssDNA independently of Cas9, thus ABE8e can edit naturally occurring ssDNA (for instance, during replication or transcription). Thus, random ssDNA editing is greatly affected by the concentration and duration of exposure to ABE8e. To eliminate these off-target edits, alternative delivery approaches were tested, such as delivering ABEs as mRNA or pre-assembled RNPs rather than plasmids, and have been shown to produce fewer off-target edits [59]. An alternative strategy could be to design ABEs where the deaminase is only active when Cas9 is bound to the proper target site, although no such ABEs have been developed yet.

Multiple-turnover enzymes like TadA8e have high processivity, which increases editing efficiency but also bystander editing [18,25]. Editing multiple adenosines within the R-loop poses a significant challenge as unintended editing of neighboring nucleotides could result in changes to the amino acid sequence. Additionally, even silent mutations may have an impact on protein function [60], abundance, and stability [61]. Currently, no ABEs are free of bystander-editing, and those with lower levels are often less efficient. It was shown that F148A reduces bystander editing of ABEmax, however it lowers the levels of on-target editing as well [62] due to its role in interacting with the DNA substrate as discussed in the Sequence recognition section (Figure 3C). Therefore, new designs are necessary to address this issue.

It is worth noting that ABEs can also deaminate deoxycytosine (dC) within ssDNA, albeit with much lower efficiency [63,64]. It was shown that Q108 reduces TadA's deoxycytosine deamination efficiency, which increases the dA/dC deamination ratio. However, N108 is one of the key residues enabling TadA to deaminate DNA (discussed in the Discrimination between RNA and DNA substrates section) [25,36]. Therefore, while Q108 lowers dC deamination, it also significantly reduces editing of the target deoxyadenosine [40].

### RNA off-target editing

In the context of ABEs, RNA off-target editing is often attributed to the characteristics of the deaminase domain. The deaminase domain in ABEs was evolved from tRNA deaminase, explaining why early versions of ABEs (where the evolved TadA and WT-TadA domains were fused to Cas9) exhibit high levels of RNA off-target editing, particularly in regions of RNA that resemble tRNA anticodon loop structures [16,17]. Efforts to mitigate RNA off-targeting in ABEs started by engineering miniABEmax, which lacks the WT-TadA domain [65]. This version of ABE showed a significant reduction in RNA off-target editing while maintaining comparable levels of DNA editing to the original ABE7.10 [17]. To further improve ABEs, multiple mutations were tested, and it was discovered that introducing either K20A/R21A or V82G mutations to the miniABEmax led to notable reductions in RNA off-targeting [17]. The precise mechanism by which K20A/R21A mutations decrease RNA off-target editing is not yet fully understood. As these residues are exposed to solvent, it is possible that mutating them to hydrophobic alanine affects the stability of TadA, but this hypothesis requires experimental validation. The V82G substitution occurs in the substrate-binding loop L4 (Figure 2A), where the smaller size of glycine, as compared with valine, may alter the loop conformation and thereby affect interactions with the substrate.

ABE8e exhibits significantly higher ssRNA off-target editing than early versions of ABEs [18]. This is because its deaminase domain discriminates between structured substrates and single-stranded ones, rather than distinguishing between DNA and RNA. As a result, it may prove difficult to engineer TadA-based ABEs with reduced off-target RNA editing while maintaining high efficiency on-target DNA editing. Therefore, there is still a need to design ABEs that can better discriminate between DNA and RNA and have lower RNA off-target editing.

Besides DNA and RNA off-target editing, ABEs are limited to editing targets within the R-loop formed by Cas effectors, restricting their editing window to a fixed distance from the specific PAM sequence. Since ABEs are modular proteins, various Cas effectors can be used to access different PAM sequences and expand the range of potential targets. Early versions of ABEs were not compatible with other Cas enzymes [16]. However, TadA8e is an exception, potentially because its deamination is faster than the off-rate of Cas effectors, resulting in deamination prior to ABEs’ dissociation from the DNA [18,25]. Huang et al. discuss in detail possible combinations of Cas effectors and deaminases, and offer insights into the best combination for a desired target [66].

## Perspectives

- DNA base editors (BEs) introduce point mutations in a programmable manner and have immense potential for exploring gene functions, treating genetic diseases, and improving agriculture. ABEs are particularly effective at converting A-to-G in genomic DNA, although the molecular basis of their specificity, precision, and efficiency remains poorly understood.
- The deaminase domain (TadA8e) of the highly efficient DNA adenine base editor (ABE8e) was obtained through the directed evolution of tRNA deaminase EcTadA. This process introduced key mutations that optimized the active site and substrate-binding loops, enabling TadA8e to distinguish between rigid, structured and flexible, single-stranded substrates, and to function as a processive and sequence non-specific deaminase.
- Several remaining questions regarding the molecular basis of ABE must be addressed to develop safe and precise genome editors for biomedical applications, including the significance of TadA8e dimerization, the mechanism of ABEs’ processivity, and how TadA8e differentiates between RNA and DNA.

## Abbreviations

ABEs: adenine base editors
BE: base editors
DSBs: double-stranded DNA breaks
HDR: homology-directed repair
MD: molecular dynamics
NTS: non-target strand
PAM: protospacer adjacent motif
SSB: single-stranded break
TS: target strand
